# Near-digital amplification in paper improves sensitivity and speed in biplexed reactions

**DOI:** 10.1038/s41598-022-18937-8

**Published:** 2022-08-26

**Authors:** Kamal G. Shah, Sujatha Kumar, Paul Yager

**Affiliations:** Department of Bioengineering, University of Washington, Seattle, WA 98195 USA

**Keywords:** Biological fluorescence, Optics and photonics, Diagnosis, Infectious diseases, Molecular imaging

## Abstract

The simplest point-of-care assays are usually paper and plastic devices that detect proteins or nucleic acids at low cost and minimal user steps, albeit with poor limits of detection. Digital assays improve limits of detection and analyte quantification by splitting a sample across many wells (or droplets), preventing diffusion, and performing analyte amplification and detection in multiple small wells. However, truly digital nucleic acid amplification tests (NAATs) require costly consumable cartridges that are precisely manufactured, aligned, and operated to enable low detection limits. In this study, we demonstrate how to implement near-digital NAATs in low-cost porous media while approaching the low limits of detection of digital assays. The near-digital NAAT was enabled by a paper membrane containing lyophilized amplification reagents that automatically, passively meters and distributes a sample over a wide area. Performing a NAAT in the paper membrane while allowing diffusion captures many of the benefits of digital NAATs if the pad is imaged at a high spatial resolution during amplification. We show that the near-digital NAAT is compatible with a low-cost paper and plastic disposable cartridge coupled to a 2-layer rigid printed circuit board heater (the MD NAAT platform). We also demonstrate compatibility with biplexing and imaging with mobile phones with different camera sensors. We show that the near-digital NAAT increased signal-to-noise ratios by ~ 10×, improved limits of detection from above 10^3^ copies of methicillin-resistant *Staphylococcus aureus* genomic DNA to between 100 and 316 copies in a biplexed reaction containing 10^5^ copies of co-amplifying internal amplification control DNA, and reduced time-to-result from 45 min of amplification to 15–20 min for the positive samples.

## Introduction

**Figure 1:**
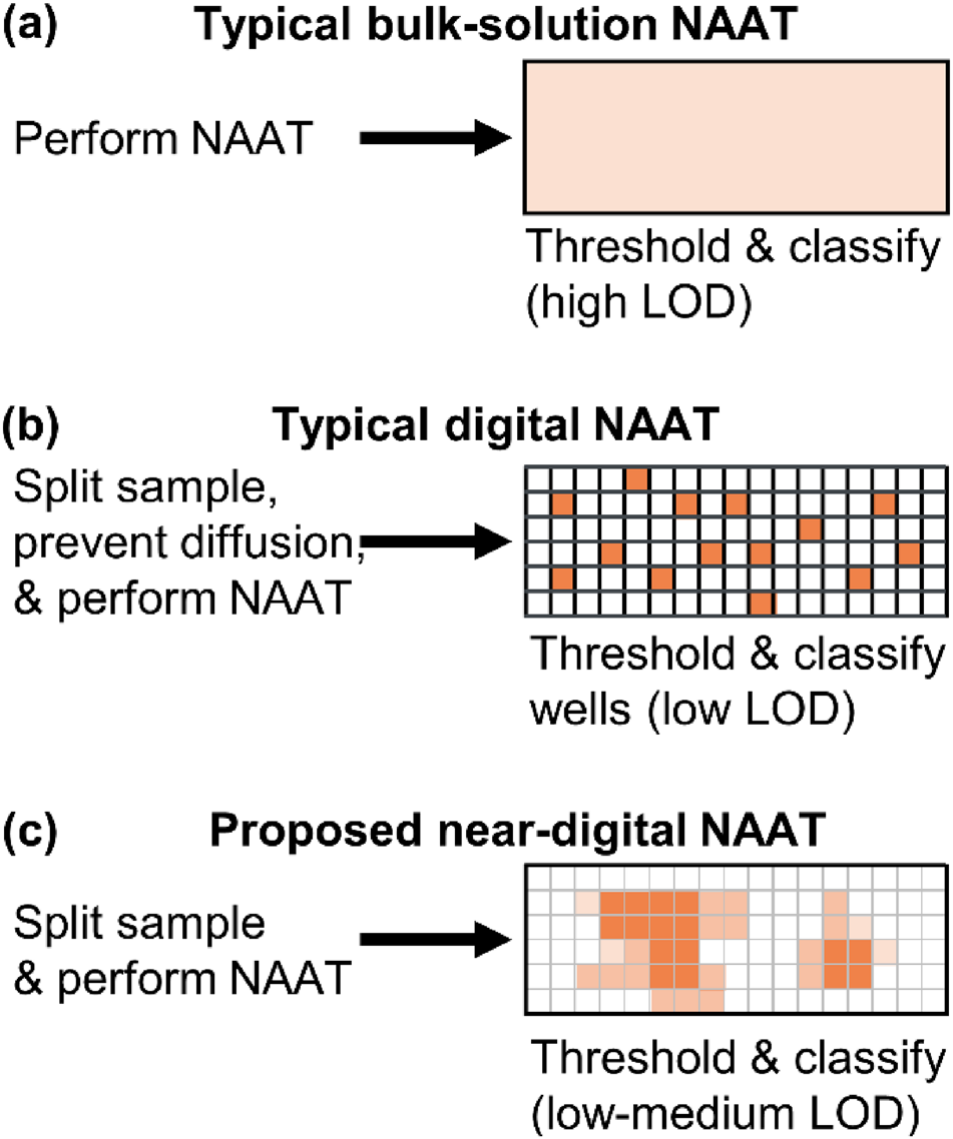
Motivation for almost-digital amplification in paper. (**a**) Typical nucleic acid amplification tests (NAATs) performed in bulk solutions have poor limits of detection (LOD) due to low spatial resolution and low initial nucleic acid concentration across the amplification zone. (**b**) Digital NAATs split the reaction mixture into multiple small wells or droplets, nominally preventing diffusion during amplification to improve limits of detection and assay time. (**c**) The near-digital NAAT initially distributes a reaction mixture across a wide area but allows diffusion during amplification, which leverages the benefits of digital NAATs without requiring complex cartridge design.

Nucleic acid amplification tests (NAATs) are part of a popular strategy to detect infectious diseases rapidly and reliably, often in integrated sample-to-result systems at the point of care^1–5^. Commercial NAATs intended for these point-of-care clinical settings usually enzymatically amplify pathogen-specific nucleic acids and detect amplification products with target-specific fluorescent probes^6–10^. These assays discriminate between the absence of target and assay failure by including onboard positive controls, often in the form of co-amplifying, heterologous internal amplification control (IAC) DNA, detected with specific probes in an orthogonal fluorescence channel (Fig. 1a)^6–9^. The combination of enzymatic amplification and multichannel fluorescence detection enable low limits of detection, rapid time to result (15 min on the semi-automated Alere i platform or 60–90 min on the automated Cepheid GeneXpert), and high specificity^6–9,11,12^.

Recent efforts to improve NAATs include many digital amplification schemes that purport to enable accurate analyte quantification, reduce limits of detection, or lower time to result^13–21^. Digital NAATs use isolated nanoliter or femtoliter scale wells or droplets to optically segment a sample into individual reaction “chambers” that contain one or zero copies of the template of interest (Fig. 1b)^15,19^. As amplification proceeds, each chamber provides a yes/no output as to whether template was present therein, and aggregating results from all chambers enables quantification under Poisson statistics. However, truly digital NAATs require a costly, precisely manufactured cartridge^13,14,16^ (for assays that amplify in wells of semi-uniform volume) or complex fluidics^19–21^ (for assays based on droplets or oil/water phase separation) to prevent diffusion between neighboring amplification chambers, which is challenging to translate to the point of care.

Several research groups have sought to reduce the complexity of digital NAATs by simplifying the underlying assumptions and required hardware. One promising approach is to perform a digital NAAT in polydisperse droplets of varying volumes, imaging the droplets with high spatial or temporal resolution, and leveraging complex statistical corrections to compensate for the variation in the sampled droplet volume^20,21^. Another droplet-based strategy is to simplify the optics by imaging droplets one-at-a-time in a format akin to flow cytometry^22^. Digital NAAT cartridges may also be simplified by laser cutting Slip-Chip-style wells in acrylic^14^. However, these approaches to date have been unsuitable to translate to the home because they require bulky/costly supporting hardware (lasers or motorized stages) or difficult to manufacture components at the nanoliter scale. In contrast, extant home assays are performed in porous media (i.e. paper microfluidics) that passively meter fluids at the microliter scale and require no complex hardware or user steps^23,24^.

Lateral flow immunoassays (e.g. pregnancy, ovulation, or HIV) are the most common paper-based assay used in the home. These assays sacrifice limits of detection for ease of use and low cost, which render them unsuitable for sensitive detection of infectious disease biomarkers^23,24^. One strategy to improve sensitivity is to perform NAATs in paper^1,3,25–27^. However, existing paper-based NAATs often lack co-amplifying positive controls and suffer from slow time to result (e.g. endpoint assays with 30–60 min of amplification time)^1,3,25–27^. We propose to overcome these limitations by leveraging insights from digital NAATs and translating them into paper microfluidic assays. Specifically, we note that if the size of the area in which nucleic acid amplification occurs is large compared to the diffusivity of the amplification products, then it is not possible for amplification in one region to affect the concentration of amplification products in a distant region (provided that the bulk fluid is not moving). In other words, the nucleic acid amplification reaction is confined to “virtual wells”, the sizes of which increase with time (based on diffusion).

The near-digital NAAT in paper leverages wicking in porous media to automatically distribute a sample over a wide surface area pad that contains lyophilized isothermal NAAT reagents. A mobile phone images the entire pad during amplification at a high spatial resolution in real time. Individual pixels of acquired images are binned into optical segments to improve signal-to-noise ratios relative to reactions performed in bulk media (Fig. 1c). The usefulness of this approach was demonstrated with an isothermal NAAT for methicillin-resistant *Staphylococcus aureus* (MRSA) performed on a portable, USB-powered NAAT platform developed previously in our lab^1,28^. The near-digital NAAT described in this manuscript is a step toward enabling rapid detection of amplifying nucleic acids in point-of-care settings, including the home.

## Methods

### Overview of near-digital NAAT

Real-time nucleic acid amplification tests usually detect amplification products optically, often with sequence-specific fluorescent probes. For a NAAT performed in a bulk solution, all components of the reaction (e.g., templates; NAAT reagents such as primers, enzymes, and nucleotides; and products) are free to equilibrate throughout the reaction volume through diffusion and convection. Diffusion kinetics are governed by Fick’s Second Law, shown in Eq. 1:

1 $$\frac{\partial C}{\partial t}=D\frac{{\partial }^{2}C}{\partial {x}^{2}}$$

where C in the concentration, D is the diffusion coefficient, t is time, and x is distance. The diffusion coefficient is a function of several variables such as particle size (based on the Stokes–Einstein equation for spherical particles) and ionic charge (Einstein–Nernst relation), which renders it difficult to accurately predict the diffusion coefficient of all species in a NAAT reaction. Moreover, in porous media, the diffusion coefficient is further modified to compensate for partitioning (K) and hydrodynamic drag (ω_r_) as in Eq. (2), where α is the hydrodynamic radius and r is the pore radius^3,29^:

2 $${D}_{m}=DK{\omega }_{r}=D{\left[1-\frac{\alpha }{r}\right]}^{2}\left[1-2.1\frac{\alpha }{r}+2.09{\left(\frac{\alpha }{r}\right)}^{3}-0.95{\left(\frac{\alpha }{r}\right)}^{5}\right]$$

Equation (2) indicates that the diffusion coefficient of most of the components of the amplification reaction in porous media should be lower than in bulk solutions since both the partition coefficient and hydrodynamic drag terms are lower than one because the pore radius (r) is almost always greater than the hydrodynamic radius of most species (α))^3,29^. This implies that NAATs performed in porous media should experience slower diffusion than assays performed in wells or droplets.

### Performing the near-digital NAAT

An isothermal strand displacement amplification (iSDA) method for the MRSA *mecA* gene target was biplexed with an internal amplification control (IAC) as previously described^1^. In a biplexed reaction, a nontarget IAC DNA sequence is present in the same reaction as the sample and is coamplified simultaneously with the target sequence. The IAC is necessary to prevent false negative results. The IAC template used for near-digital NAAT was a synthetic double-stranded DNA that is flanked by primer binding sites that matched the *mecA* target primer sequence, and detected by its interior-sequence-matching probe. The probe for IAC is labeled with a different fluorophore than the *mecA* target detection probe. This approach enables co-amplification of the IAC using the same target primers thus eliminating the need to add extra primer sets for the IAC in the biplexed reaction.

The near-digital NAAT assays were performed in a Whatman Standard 17 glass fiber membrane. The membrane is ~ 370 µm in thickness and the pore features are over a large length scale ~ 1 to 50 µm (as observed by scanning electron microscope published in Kumar et al*.*^30^ Supplementary information online). The procedure for iSDA amplification in the porous membrane is outlined in our previous publications^1,30^. Briefly, glass fiber membranes were laser-cut (VLS3.60, Universal Laser Systems, Scottsdale, AZ) to 15 mm × 3 mm strips having a fluid capacity of 20 µl, blocked with 1% BSA, 0.1% Tween 20, filled with all reagents (including enzymes and primers) for iSDA, flash frozen with liquid nitrogen, lyophilized for at least two hours, and stored in heat-sealed moisture barrier foil pouch (Ted Pella Inc., Redding, CA) at room temperature until use.

The baseline (1×) iSDA chemistry consisted of: inorganic potassium phosphate buffer (pH 7.6, 50 mM), magnesium sulfate (3.75 mM), an equimolar mix of deoxynucleotides [dATP, dCTP, dGTP, dTTP] (0.2 mM), trehalose (0.27 M), 500 kD dextran (4.8% (w/v)), primers for the MRSA *mecA* gene target (500 nM forward, 250 nM reverse, 50 mM of each bumper primers), target-specific fluorescent probes (minor groove binders from ELITechGroup) labeled with a Texas Red analog (AquaPhluor-593, 200 nM), internal amplification control-specific probes labeled with 6-FAM (200 nM), Bst 2.0 Warmstart DNA polymerase (New England BioLabs, 200 U/µL), Nt.BbvC1 nicking enzyme (New England BioLabs, 1.6% (v/v)), and nuclease-free water (1.23 µL). The sequences for iSDA primers for the *mecA* gene target are given in Supplementary Table S1 online.

The pads were rehydrated with a 20 µL mixture of methicillin-resistant *Staphylococcus aureus* (MRSA) strain FPR3757 genomic DNA (ATCC BAA-1556DQ) and 10^5^ copies of internal amplification control (IAC) (sequence for IAC is given in Supplementary Table S1 online) in nuclease-free water. The pads were placed in laser-cut polymethylmethacrylate cartridges and were covered with a plastic film that is impermeable to water/water vapor during amplification to minimize evaporation. The cartridges where then adhered to the printed circuit board of the USB-powered multiplexed, disposable NAAT (MD NAAT) device (described in the PhD thesis of K. G. Shah, available from the University of Washington) using thermally conductive tape (3M 8815)^31^. An image of the MD NAAT device setup is shown in Supplementary Figure S1. The pads were heated to iSDA-relevant temperatures (50 °C) by the MD NAAT device for 45 min. A mobile phone fluorescence reader (Nexus 5X or Pixel 2 equipped with multipass excitation and emission filters) detailed in Shah et al.^28^ was placed about 15 cm above the MD NAAT and imaged the pads every 30 s using the Camera FV-5 application.

### Optically segmenting mobile phone images

Mobile phone images were preprocessed by gamma correcting, rotating, and cropping them to the region corresponding to each pad. Each glass fiber amplification pad corresponded to about 5 × 10^4^ to 10^5^ pixels on the phones used. The differential fluorescence intensity (difference of red and green color channels) was calculated at each pixel. The pads were segmented into blocks by grouping neighboring pixels such that each pad contained 1, 4, 10, 100, 1000, 10^4^, or the maximum possible number of blocks (i.e., the number of pixels in the region of interest). The nominal block dimensions are shown in Table 1. The average differential fluorescence intensity was then calculated for each block, and each pad’s intensity was defined as the average of the top decile of intra-block intensities (i.e., the average of the 10% highest blocks, which is an approach for truly digital NAATs identified by Rolando et al. as balancing the competing needs to maximize sensitivity and minimize false positives)^18^. All analysis was performed in MATLAB R2019a.

**Table 1:** Nominal block parameters in optically segmented mobile phone images.

Blocks	Blocks in 3 × 15 mm pad	Block dimensions* (µm)	Block area	Nominal block volume* (for 20 µL reaction)
1	1 × 1	3000 × 15,000	45 mm^2^	20 µL
4	1 × 4	3000 × 3750	11.3 mm^2^	5 µL
10	2 × 5	1500 × 3000	4.5 mm^2^	2 µL
100	4 × 25	750 × 600	0.45 mm^2^	200 nL
1000	20 × 50	150 × 300	45,000 µm^2^	20 nL
10,000	40 × 250	75 × 60	4500 µm^2^	2 nL
100,000	200 × 500	15 × 30	450 µm^2^	0.2 nL

*The nominal block volumes are based on distributing a 20 µL sample volume across the number of blocks in the first column. This calculation does not make any assumptions about the thickness of the amplification zone, which is affected not only by the thickness of the porous media used, but also by the volume of the amplification reagents and surface effects (e.g., contact angle).

### Validating near-digital NAAT

#### Assess improvement of signal-to-noise ratios

First, we validated whether optical segmentation improved signal to noise ratios in the near-digital NAAT. Glass fiber pads containing lyophilized iSDA reagents for the MRSA *mecA* gene were rehydrated with biplexed mixtures of MRSA genomic DNA and IAC DNA, heated in the MD NAAT, and imaged with the Nexus 5X fluorescence reader. The images were analyzed as in the previous paragraph. A one-way analysis of variance (ANOVA) was used to assess whether the endpoint differential fluorescence at 45 min was significantly different between MRSA-positive pads containing 1000 copies of MRSA genomic DNA mixed with 10^5^ copies of IAC DNA and MRSA-negative pads containing 10^5^ copies of IAC DNA.

#### Characterizing the impact of diffusion

Next, we assessed the impact of diffusion in the near-digital NAAT. We rehydrated lyophilized iSDA reagents (which contained red-emitting fluorescent probes specific to the MRSA *mecA* gene) with 10^6^ copies of a synthetic, single-stranded DNA (ssDNA) oligomer containing the *mecA* gene. The pads were heated in the MD NAAT (to 50 °C) and imaged with the mobile phone fluorescence reader with a Nexus 5X or Pixel 2 phone about every 30 s (n = 4 pads total). Positive and negative controls were simultaneously heated for 45 min in a heat block set to 50 °C and imaged with the phone reader at the endpoint to verify that contamination did not occur.

Images of the MD NAAT runs were analyzed with a computer vision algorithm that quantified propagation of the amplification front. The red channel of acquired images was binarized by thresholding, and the regionprops() function was used to calculate the equivalent diameter of a circle with the same area as the region above the threshold. These diameters were compared to the expected propagation of the amplification front based on diffusion in porous media, i.e., the one-dimensional, semi-infinite solution to Eq. (1), whereby the position of the propagation front is proportional to the square root of the modified diffusion coefficient and elapsed time.

#### Optimizing the near-digital NAAT

Next, we optimized the near-digital NAAT chemistry to maximize signal-to-noise ratios in biplexed isothermal strand displacement amplification reactions. NAAT chemistry optimization was guided by fractional factorial experiment design principles by varying the concentration of the 5 reagents that were previously implicated in affecting iSDA time to result in tube reactions. In the first experiment, 18 master mixes were prepared by varying the nicking enzyme (0.5×, 1×, 1.5×), polymerase (1×, 1.5×, 2×), and primers (1×, 1.5×) concentrations. The master mixes were lyophilized onto blocked glass fiber pads as above. A custom pad holder was engineered from 3 mm-thick clear PMMA by mimicking the shape of a standard 384-well plate and having laser-etched regions for each lyophilized pad such that each 3 × 15 mm pad aligned with 4 wells of a well plate. Each pad holder region was treated with dichloromethane (23 µL, corresponding to 0.5 µL/mm^2^) and the bottom of the PMMA tray was covered with black LDPE secured by minimally fluorescent PDMS tape. Lyophilized pads were placed on the custom PMMA tray and were reconstituted with 1000 copies of genomic DNA purified from MRSA genomic DNA mixed with 10^5^ copies of single-stranded IAC DNA in 20 µL of nuclease-free water. The tray was placed in a preheated plate reader (Molecular Devices) set to 50.5 °C, configured to read a 384-well plate, and imaged in fluorescence kinetic mode (excitation: 593 nm, emission: 650 nm, 1 s integration time, about 5 min to read the entire plate) over about 1 h. In the second experiment, 18 master mixes were evaluated by testing three of the optimal master mixes identified from the first experiment at a range of concentrations of dNTPs (1×, 1.5×, 2×) and magnesium sulfate (1×, 1.5×) (procedure identical to the first experiment). Both experiments were run with triplicate pads at each condition along with a set of positive and negative controls (i.e., sans MRSA DNA) rehydrating the baseline (1×) master mix.

Finally, the near-digital NAAT was run at the optimized reagent concentrations identified in the previous two experiments. Lyophilized pads containing iSDA reagents were rehydrated with a dilution series of mixtures of MRSA genomic DNA and IAC ssDNA, heated in the MD NAAT for 45 min, and imaged with a Nexus 5X mobile phone fluorescence reader for 45 min (n = 3). Images were analyzed with and without the near-digital optical segmenting scheme outlined previously. The time to threshold (set at 0.02 a.u. for MRSA-positive reactions and − 0.02 a.u. for MRSA-negative reactions) was quantified for both the segmented and unsegmented cases. A one-sample, parametric t-test assessed whether there was a significant difference in the time to result.

## Results

### Assessing improvement to signal-to-noise ratios

**Figure 2:**
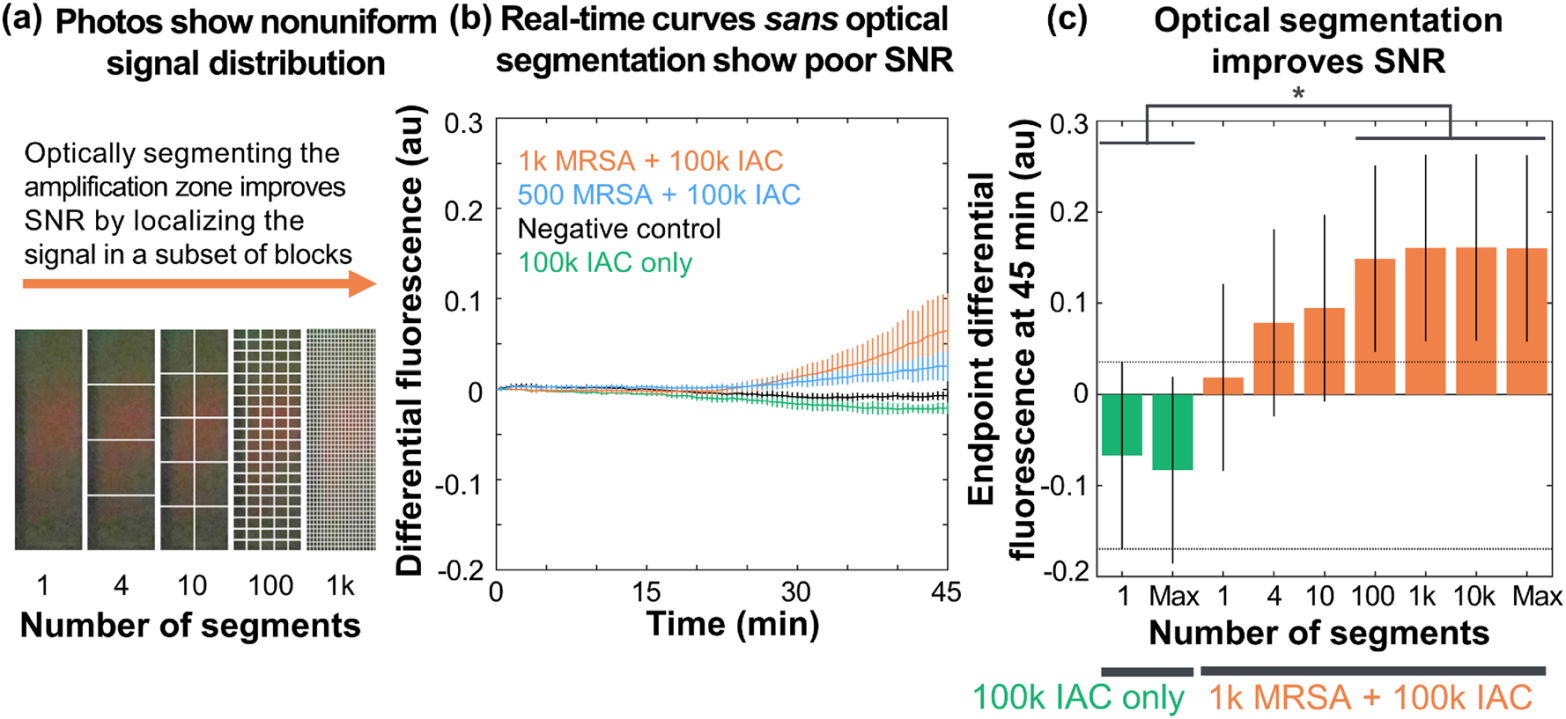
Optical segmentation improves signal-to-noise ratios of nucleic acid amplification in porous media. (**a**) A photo (with differing densities of overlaid lines) of a biplexed amplification reaction containing MRSA DNA and internal amplification control (IAC) DNA shows a highly nonuniform signal distribution after 30 min of amplification. Optically segmenting the pad and analyzing a subset of blocks isolates the signal of interest, improving signal-to-noise ratios. (**b**) Real-time curves for biplexed isothermal strand displacement amplification reactions imaged with a mobile phone fluorescence reader show slow liftoff times and poor signal-to-noise ratios in unsegmented pads. Curves show mean and error bars showing the standard error of the mean (n = 5). (**c**) Plotting the endpoint signal intensities for the IAC-only and MRSA-positive biplexed reactions shows a significant improvement in signal-to-noise ratios. The endpoint differential fluorescence was significantly greater in MRSA-positive pads optically segmented into at least 100 blocks than the IAC only pads (p < 0.05, one-way ANOVA). The y-axes in both panels (**b**,**c**) show the differential fluorescence intensity (i.e., difference of red and green color channels).

First, we assessed whether near-digital nucleic acid amplification in glass fiber membranes (with optical segmentation) improved signal-to-noise ratios compared to unsegmented reactions. Mixtures of MRSA genomic DNA and IAC DNA were diluted in buffer, amplified by isothermal strand displacement amplification (iSDA) in the MD NAAT device, and imaged every 30 s by a Nexus 5X. Figure 2a shows that images of a biplexed reaction containing 1000 copies of MRSA mixed with IAC DNA show nonuniformly distributed red, MRSA-specific fluorescence near the center of the pad after 30 min of amplification. The pad also showed substantial green fluorescence near the edges of the pad. Increasing the number of segments into which the image was divided isolated the red fluorescence of interest into a subset of blocks.

Next, we quantitatively analyzed the images from this experiment, but without optical segmentation. The real-time curves in Fig. 2b show that data from pads as a whole (*sans* optical segmentation) have low signal-to-noise ratios, even after 45 min of amplification (n = 5). The negative controls were indeed negative: pads containing neither MRSA nor IAC DNA were in the range corresponding to no amplification [i.e., above the threshold for MRSA-negative (p < 0.01, right-tailed one-sample t-test) and below the threshold for MRSA-positive (p < 0.001, left-tailed one-sample t-test)] at all times. However, without optical segmentation, pads containing only IAC DNA or both MRSA and IAC DNA did not significantly exceed the threshold for negative or positive, respectively (p > 0.05, one-sample t-test).

Finally, the images from this experiment were analyzed with the near-digital optical segmentation scheme. Figure 2c demonstrates that increasing the number of blocks (to 100) into which each pad was optically segmented improved signal-to-noise ratios: pads containing 1 k copies of MRSA (with 100 k copies of IAC) significantly exceeded the threshold for positive (p < 0.05, one-sample t-test) and were significantly greater than the IAC only group (p < 0.05, one-way ANOVA). However, increasing the spatial resolution beyond 100 blocks (i.e., decreasing the block size below 750 × 600 µm) did not significantly improve endpoint signal-to-noise ratios. There was no significant difference in endpoint fluorescence intensities for unsegmented pads (p > 0.05, one-way ANOVA).

### Characterizing impact of diffusion

**Figure 3:**
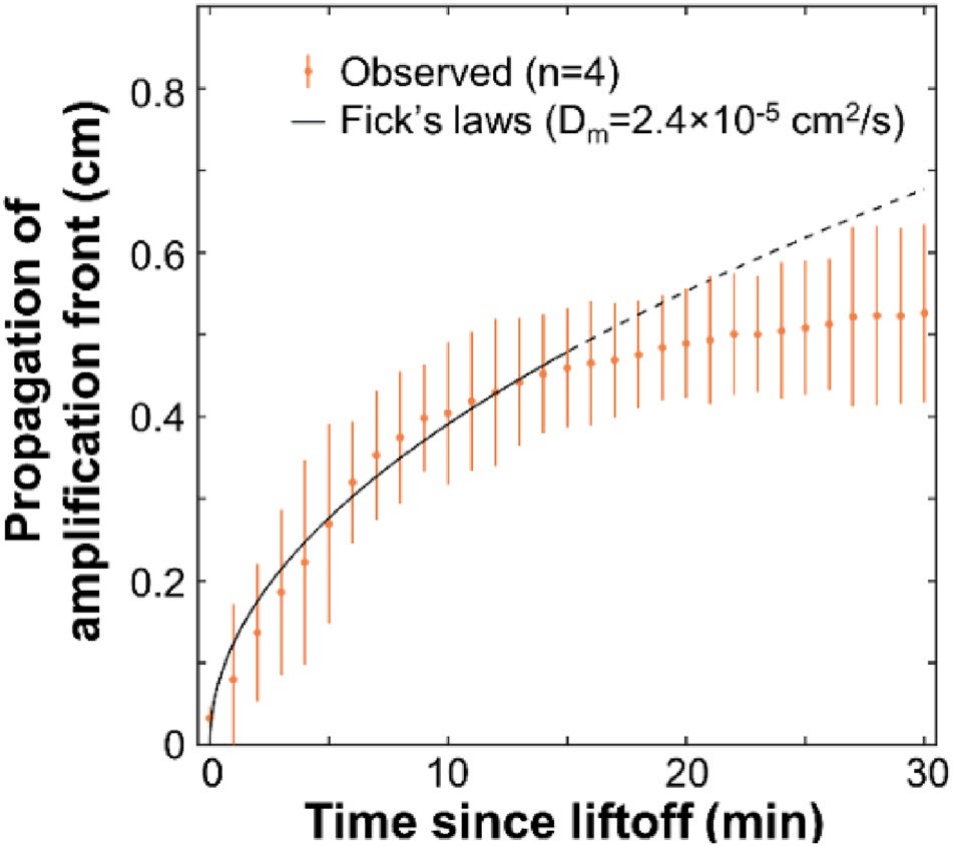
Propagation of amplification front follows Fick’s laws immediately after liftoff. Glass fiber pads containing lyophilized iSDA reagents were imaged after being rehydrated with 10^6^ copies of a synthetic single-stranded DNA oligomer containing the *mecA* gene of MRSA (n = 4 pads, 2 each on the Nexus 5X and Pixel 2 phones). The real-time images were gamma-corrected, thresholded in the red color channel, and analyzed by a computer vison algorithm detailed in the text accompanying this figure. The y-axis shows the equivalent diameter of a circle with the same area as the pixels above the threshold, while the x-axis shows the time elapsed since amplification lifted off. The propagation of the amplification front followed Fick’s laws for the first 15 min after amplification (solid black line), with a modeled modified diffusion coefficient (D_m_) of 2.4 × 10^–5^ cm^2^/s (p < 10^–13^, one-sample t-test). However, propagation afterward is significantly lower than diffusion as shown by the minimal overlap between the dashed black line and error bars. Points show mean and error bars show 95% confidence intervals.

We then characterized propagation of the amplification front during the near-digital NAAT since species are free to diffuse during amplification. Pads containing lyophilized iSDA chemistry were rehydrated with single-stranded DNA containing the *mecA* gene, imaged with two phones during amplification, and analyzed with a computer vision algorithm to track propagation of the amplification front (n = 4 pads). Figure 3 shows that the amplification front expanded following Fick’s laws of diffusion during the first 15 min after amplification had lifted off (root mean squared error = 0.03 cm). The fitted modified diffusion coefficient (D_m_) was 2.4 × 10^−5^ cm^2^/s (p < 10^–13^, one-sample t-test) during the first 15 min after liftoff. The amplification front propagated slower than diffusion after 15 min post-liftoff, as indicated by the minimal overlap between the dashed black line and 95% confidence intervals in Fig. 3. The positive and negative controls that were run in a heat block and imaged with a mobile phone at the endpoint indeed showed red fluorescence (indicating amplification in the positive controls) and no fluorescence (indicating no amplification in the negative controls), respectively (n = 2) [photos not shown].

### Optimizing near-digital NAAT

The previous two experiments suggested that the assay had low signal-to-noise ratios, which we hypothesized could be improved by adjusting the assay chemistry. Therefore, we optimized the lyophilized master mix to reduce the time to result (and improve signal-to-noise ratios) by performing high throughput, paper-based iSDA experiments in a plate reader. First, we optimized the concentration of polymerase, nicking enzymes, and primers by testing 18 master mixes in biplexed amplification reactions. Supplementary online Figure S2a shows that 7 master mixes rehydrated with MRSA-positive samples showed significantly higher MRSA-specific fluorescence at 30 min than the negative control (p < 0.05, one-way ANOVA). Three of these master mixes were evaluated at a range of nucleotide and magnesium sulfate concentrations (see Supplementary Fig. S2b online). One master mix (containing 1.5 × the baseline levels of polymerase, nicking enzymes, and magnesium sulfate; 2 × the baseline levels of nucleotides, and 1 × the baseline levels of primers) amplified by 25 min by having significantly higher MRSA-specific fluorescence relative to the negative control (p < 0.05, one-way ANOVA).

### Demonstrating shorter time to positive with optical segmentation

**Figure 4:**
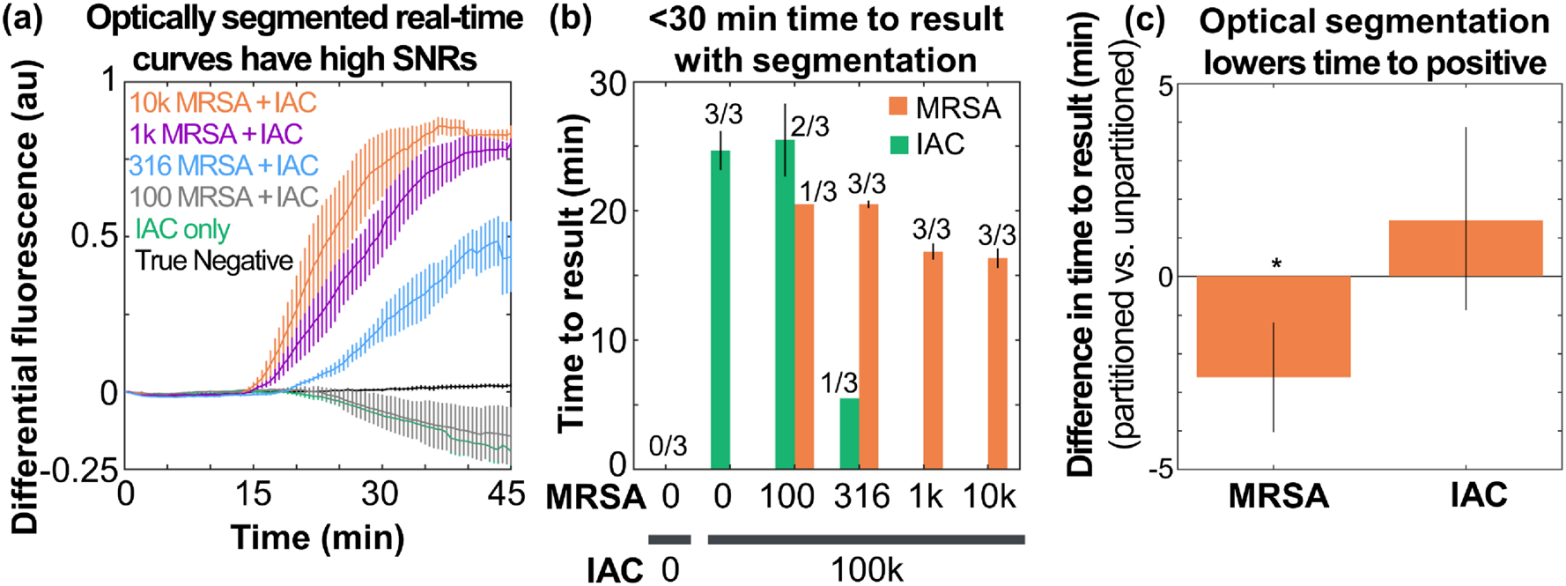
Demonstrating improved signal-to-noise ratios and time to result with near-digital NAATs. Pads containing lyophilized master mix at the optimized reagent concentrations were rehydrated with MRSA DNA biplexed with 100 k copies of internal amplification control (IAC) DNA. (**a**) Real-time curves with optical segmentation show rapid amplification and high signal-to-background ratios for both MRSA positive and MRSA negative samples with no false positives. (**b**) The time to result for the MRSA channel is about 15–20 min in MRSA-positive samples above the limit of detection and 25 min for the IAC channel below the limit of detection. (**c**) Optical segmentation lowers the time to result in the MRSA channel by 2.6 min relative to unsegmented pads (p < 0.05, one-sample t-test), but without significantly changing the time to negative (p > 0.05, one-sample t-test). In all panels, curves and bars show mean and standard error of the mean for n = 3 pads.

Finally, we evaluated the near-digital NAAT with the optimizing chemistry using biplexed samples containing MRSA genomic DNA mixed with 100 k copies of IAC. Real-time curves showed rapid, reproducible amplification; MRSA-positive samples at 316 copies and above showed positive differential fluorescence signals and IAC only samples consistently had negative differential signals (Fig. 4a). Times to positive were 16.3 ± 2.3 min (mean ± std. dev.) at 10 k copies of MRSA, 16.8 ± 1.9 min at 1 k copies, and 20.5 ± 0.9 min at 316 copies. Times to negative were 24.7 ± 2.6 min (mean ± std. dev.) for the IAC only controls (Fig. 4b). One of three samples containing 100 copies of MRSA reached the threshold for positive in 20.5 min, whereas the other two samples reached the threshold for negative in 25.5 min on average (Fig. 4b). An example video of the near-digital NAAT showing real-time curves and corresponding images for a MRSA-positive at 316 copies with 100 k copies of IAC, 100 k copies of IAC, and a true negative is provided in the Supplementary information online. Gel electrophoresis confirmed that no MRSA or IAC-specific bands appeared in the truly negative samples, but that amplification did indeed occur (see Supplementary Fig. S3 online). Figure 4c shows that the time to positive was 2.6 min lower using the near-digital optical segmentation scheme than in unsegmented pads (p < 0.05, one-sample t-test), but the time to negative did not change significantly (p > 0.05, one-sample t-test).

## Discussion

Digital assays are a promising class of NAATs that partition an amplification reaction into multiple zones to improve time to result and limits of detection. Truly digital NAATs quantify nucleic acids based on Poisson statistics by assuming that individual wells or droplets are completely isolated. In practice, truly digital NAATs are often relegated to laboratory use due to the need for complex disposable cartridges based on traditional or droplet microfluidics. However, many digital NAATs still suffer from channeling and diffusion between neighboring amplification zones^18^. Recently, Sullivan et al*.* reported recombinase polymerase amplification in paper membranes where amplification nucleation sites were quantified and correlated to input copy numbers per reaction^32^. This manuscript describes a step toward translating digital NAATs to the point of care by implementing near-digital optical segmentation of a stationary (non-flowing) amplification reaction in a porous matrix. The strategy consists of performing a NAAT in porous media (while allowing diffusion) and imaging at high spatial resolution with a mobile phone.

The near-digital NAAT captures many of the benefits of truly digital NAATs with far simpler fluidics and assay design. Porous media automatically and passively wick fluids without pumps and enable storing lyophilized amplification reagents in situ^23^. Successfully implementing a near-digital assay in paper requires overcoming several constraints imposed by paper microfluidics: (1) optical artifacts caused by evaporation; (2) autofluorescence^33^; (3) low signal-to-noise ratios; and (4) limitations of low-cost hardware to uniformly heat a low thermal mass, high surface area region during amplification. These concerns were mitigated by a combination of algorithmic, assay, and hardware optimizations advanced by our group.

Real-time imaging of the near-digital NAAT was enabled by a smartphone fluorescence reader, which has high spatial (up to 80,000 pixels per pad (~ 560 µm^2^/pixel)) and temporal resolution (every 30 s) over a wide field of view (up to 5 × 5 cm). We observed that the benefits of high spatial resolution imaging diminished when pads were optically segmented to greater than 100 blocks (750 × 600 µm, or 200 nL each) as shown in Fig. 2. We hypothesize that this was due to diffusion of amplification products between neighboring blocks: the observed modified diffusion coefficient of 2.4 × 10–5 cm^2^/s (Fig. 3) implies that the amplification front would take 68 min (greater than the assay time) to traverse the 3 mm pad width (e.g. for an unsegmented pad), 2.7 min to travel the 600 µm block width when a pad is segmented into 100 blocks, or only 10 s to traverse 150 µm block width for a pad segmented into 1000 blocks.

The simplified diffusion model provides two insights toward optimizing the near-digital NAAT in the future. First, performing the near-digital NAAT in a porous membrane with a small pore radius should decrease effective diffusion coefficients based on Eq. (2). Higher amplification speeds (e.g. by using a faster enzyme such as Bst 3.0) may also reduce the adverse impact of diffusion by generating detectable amplification products more quickly (before they diffuse away). For example, we observed that the assay chemistry optimizations shown in Figure S2 reduced liftoff times by about 15 min and improved endpoint differential fluorescence levels by a factor of 10 (between Figs. 2 and  4).

One important observation was that the modified diffusion coefficient of 2.4 × 10^–5^ cm^2^/s calculated in Fig. 3 is substantially greater than expected. For example, a DNA fragment of 50 bp has a nominal diffusion coefficient of 2.9 × 10^–7^ cm^2^/s in a bulk aqueous solution^34^. This 80-fold disparity is likely due to three main factors. First, there may have been heating-induced mass transport during amplification, driven by thermal gradients since the pads are only heated from below. Indeed, the Prandtl number for water at 50 °C is about 3.6 [and is even higher for viscous solutions (e.g. NAAT reagents)], which implies that advection of the amplification products and/or iSDA reagents could have skewed the calculated diffusion coefficient higher. Second, heating during amplification likely concentrated the amplification products as the aqueous solvent evaporated. Third, and most importantly, the amplification front could have propagated from one block to a neighboring region not only by diffusion but also by amplification (i.e. biochemical reaction) in that neighboring region. In other words, rapid amplification in one region increases the concentration gradient (and diffusional flux) of the amplification products to neighboring areas, which would appear as faster-than-diffusion transport. In future work, fully accounting for all three factors in a diffusion–advection–reaction model could yield valuable insights to the true diffusion coefficient and potentially guide future optimizations of the near-digital NAAT.

The near-digital assay with optically segmented mobile phone imaging described in this manuscript has several advantages over standard assays that lack segmentation. This was enabled in part by integrating the recent algorithmic^28^, assay, and hardware innovations (that can be found in the PhD thesis of K. G. Shah, available from the University of Washington)^31^. Optical segmentation increased signal-to-noise ratios by ~ 10 × (without leading to false positives or false negatives by following the principles outlined by Rolando et al.^18^), improved limits of detection (from above 1000 copies of MRSA genomic DNA to between 100 and 316 copies in biplexed reactions), and reduced time to result (from 45 min of amplification to 15–20 min for positive samples and 25 min for negative samples). These results were generalizable to multiple assays (e.g., *mecA* and *ldh1* genes for MRSA)^28^, phone models with vastly different camera spectra (e.g. Nexus 5X and Pixel 2), and more complex samples (e.g. MRSA cells lysed in the MD NAAT as described in the thesis)^31^.

Table 2 shows how the near-digital NAAT compares with digital and point-of-care NAATs in the literature. The near-digital NAAT has 10-min faster amplification times (and 25 min faster times to result) and lower limits of detection than the biplexed colorimetric lateral flow NAAT that we developed previously^1^. Limits of detection are lower than in bulk-solution assays^3,14^, but higher than truly digital (albeit singleplexed) assays^13^. This remarkable performance was largely enabled by the low-cost paper substrate (< $0.05) which stored lyophilized amplification reagents, passively metered and transported samples, and distributed samples over a wide area (thereby limiting the effects of diffusion). These results suggest that phone-imaged near-digital NAATs in paper are a promising step toward translating digital NAATs to the point of care. Future work from our group will report on implementing near-digital NAATs using complex samples, such as the nasal matrix, in a sample-to-result POC device.

**Table 2:** Representative digital and point-of-care NAATs.

Operating principle	Readout	Consumable*	Instrument*	Point of care?	Limit of detection	References
Nondigital recombinase polymerase amplification in 500 nL wells	Fluorescence (singleplex)	PMMA SlipChip with laser-cut and CNC-milled features	Precision alignment tools (linear stage and micrometer drive) and reader (laser, filters, and photomultiplier tube)	No	1000 copies in buffer, 20 min of amplification time	^14^
Digital loop-mediated amplification in < 10 nL wells	Colorimetric (singleplex)	Glass SlipChip fabricated with photolithography	Mobile phones with particular camera sensor	No	< 40 copies in buffer, 50 min of amplification	^13^
Nondigital loop-mediated amplification in paper	Lateral flow (colorimetric, singleplex)	Paper and plastic laminate ($2.25)	Mobile phone (or eye) and custom heater ($70)	Yes	3 × 10^5^ copies of HIV viral particles in water or whole blood, 60 min of amplification	^3^
Nondigital isothermal strand displacement amplification in paper	Lateral flow (colorimetric, biplex)	Paper and plastic with laser-cut features	Mobile phone or eye	Yes	5000 copies per device (600 copies per pad) in nasal swabs, 30 min of amplification + 15 min lateral flow readout (45 min to result)	^1^
Near-digital isothermal strand displacement amplification in paper	Fluorescence (biplex)	Paper and plastic with laser-cut features	Any mobile phone, optical filters, and disposable heater	Yes	316 copies in buffer in biplexed reactions in 20.5 min of amplification	This work

*Prices, where indicated, are bill of materials costs sans labor.

## Supplementary Information

Supplementary Video 1.

Supplementary Information 1.

## Data Availability

All data are available in the main text or the supplementary materials.
