# Supplementary material for: Near-digital amplification in paper improves sensitivity and speed in biplexed reactions
Source: Sci Rep. 2022 Aug 26;12:14618. doi: 10.1038/s41598-022-18937-8 (PMC9418329; doi:10.1038/s41598-022-18937-8)
Supplement: Supplementary file 2 — Supplementary Information 1. [file 41598_2022_18937_MOESM2_ESM.pdf]

**Supplementary information**

# Near-digital amplification in paper improves sensitivity and speed in biplexed reactions

Kamal G. Shah, Sujatha Kumar*, and Paul Yager

Department of Bioengineering, University of Washington, Seattle, WA, 98195.

**Table S1. Isothermal strand displacement assay (iSDA) nucleic acid sequence designs for *mecA* primers and internal amplification control (IAC) template.**

| | |
|---|---|
| Forward amplification primer (F) (CCTCAGC = nicking site) | 5′–CCA TTA TAC TAC CTG TCT <u>CCTCAGC</u> GGC AAA GAT ATT CAA CTA AC–3′ |
| Reverse amplification primer (R) (CCTCAGC = nicking site) | 5′–TAG AAT AGT CAC TTA CTT <u>CCTCAGC</u> GCC ATA ATC ATT TTT CAT GTT–3′ |
| Forward bumper primer (FB) | 5′–GAT AAT AGC AAT ACA ATC GCA CA–3′ |
| Reverse bumper primer (RB) | 5′–GTG CTA ATA ATT CAC CTG TTT GA–3′ |
| Internal amplification control (IAC) (dsDNA template) | 5′–GAT AAT AGC AAT ACA ATC GCA CAT GGC AAA GAT ATT CAA CTA ACG ACC AGT TAC TTT ACG GAC CAC GTA CCG CAT TGG TAC AAG ATC TCC AAC ATG AAA AAT GAT TAT GGC TTC AAA CAG GTG AAT TAT TAG CAC–3′ |

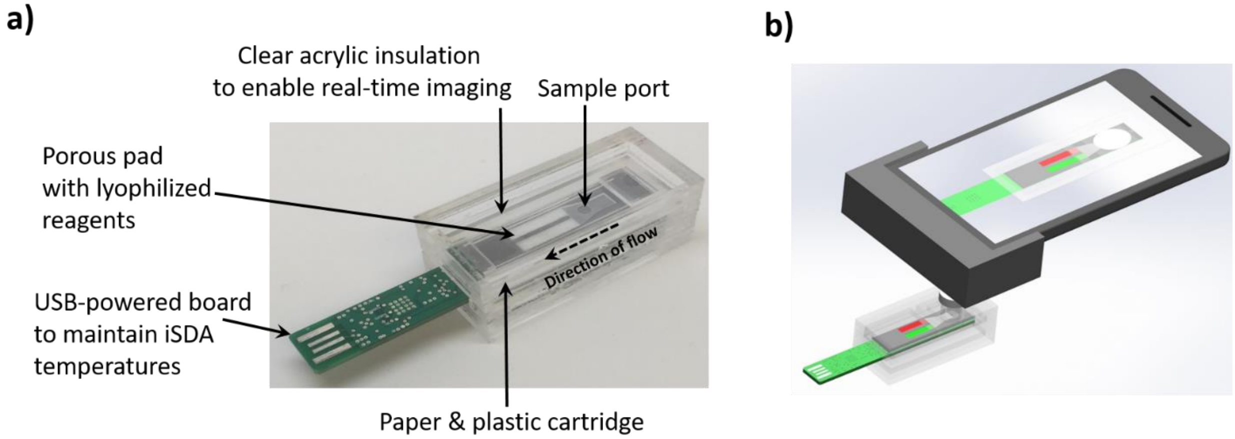


**Figure S1**. **MD NAAT platform for imaging real-time amplification.** (a) A photo of plastic cartridge holding the lyophilized iSDA reagents in porous pads. The cartridge is attached to a USB-powered PCB board. (b) A schematic of the two-fluorophore mobile phone reader for imaging MD NAAT device.

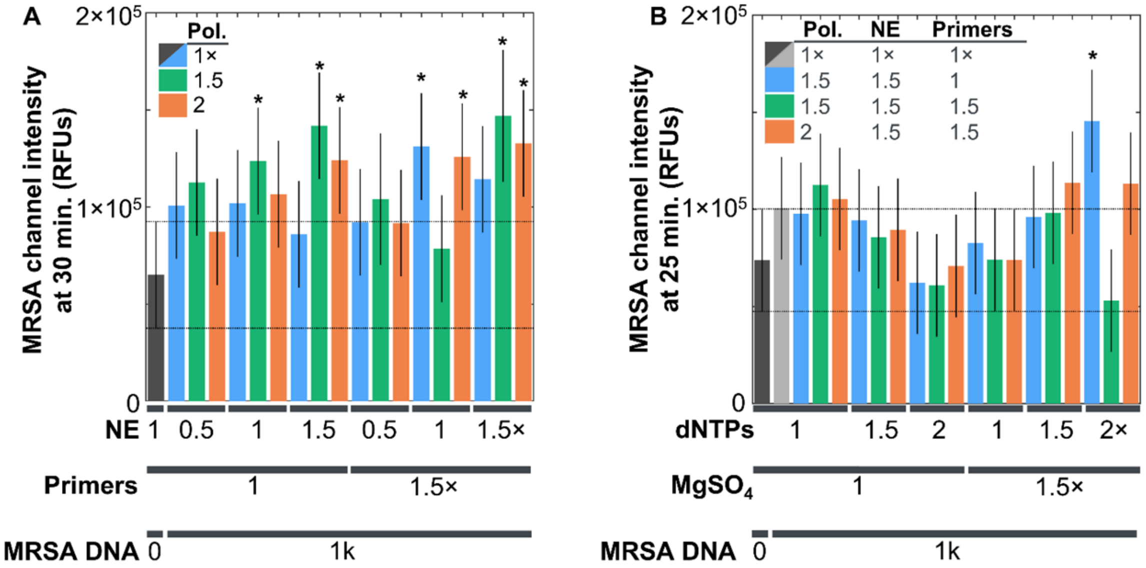


**Figure S2**. **Optimizing assay time to result** by varying the lyophilized master mix concentrations of polymerase (pol.), nicking enzyme (NE), primers, nucleotides (dNTPs), and magnesium sulfate ($MgSO_4$) relative to baseline (1×). All master mixes were lyophilized in glass fiber pads, rehydrated with MRSA DNA mixed with 100k copies of internal amplification control DNA, and run in a PMMA tray in a heated fluorescence plate reader. (**a**) Seven master mixes amplified by 30 minutes. (**b**) Three of the master mixes from Panel A were further tested with varying levels of nucleotides and magnesium sulfate. One master mix amplified by 25 minutes. In both panels, bars show mean and 95% confidence intervals for 3 replicates (* indicates $p<0.05$, one-way ANOVA relative to negative control).

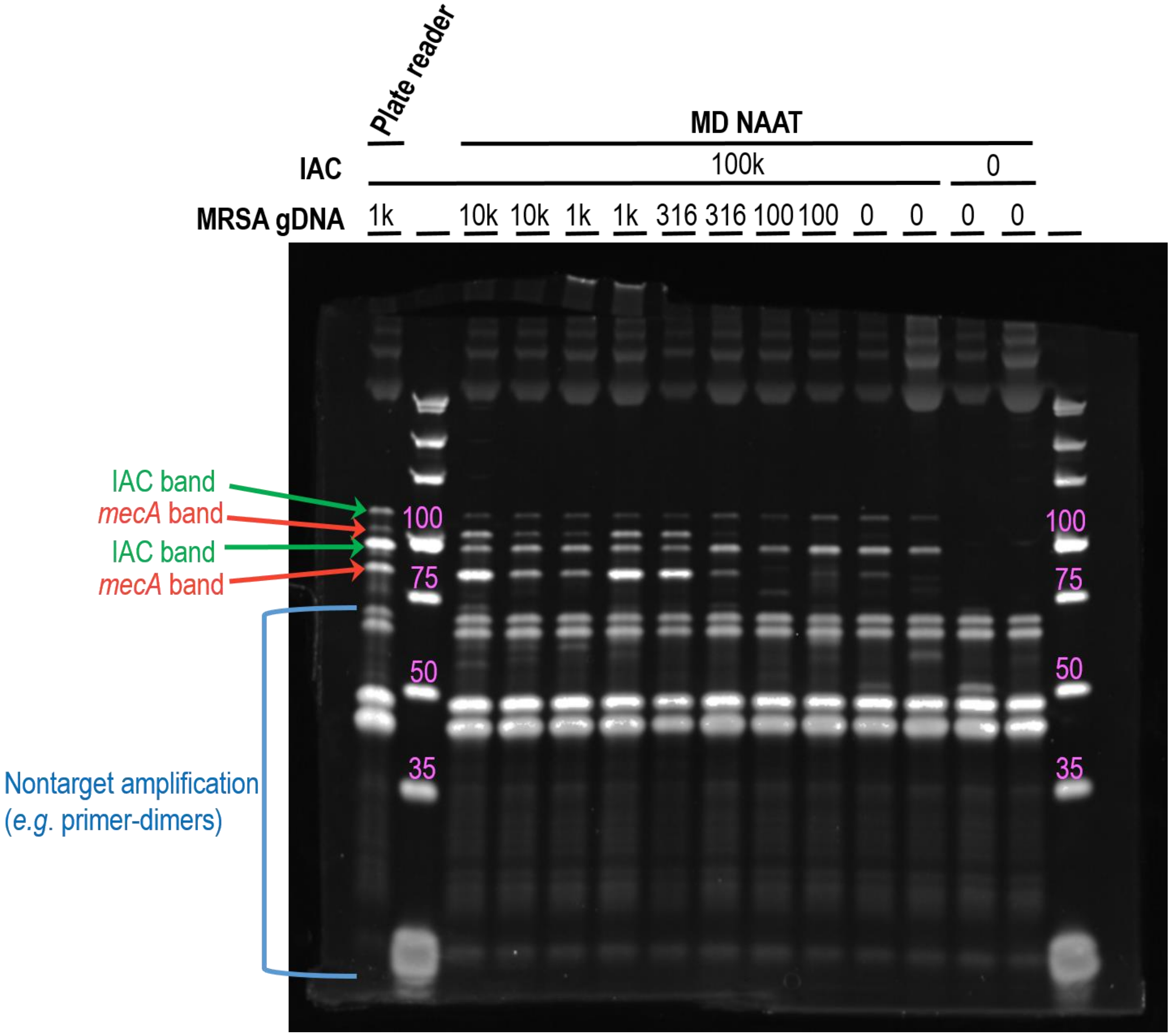


**Figure S3. Gel electrophoresis of completed amplification reactions** which were run in paper and centrifuged to isolate the liquid in the amplification pads corroborate real-time phone images. Amplification in glass fiber pads in the MD NAAT show amplification that was similar to a tube control run in a plate reader (far left). Bands corresponding to the *mecA* gene and internal amplification control (IAC) DNA were observed. The particular isothermal amplification strategy, isothermal strand displacement amplification, always shows bands from nontarget amplification (*e.g.* primer-dimers) such as those shown in the lower half of the gel. Truly negative reactions were indeed negative, all reactions containing IAC showed IAC-specific bands, and all MRSA-containing reactions showed *mecA*-specific bands at above 316 copies.

**Supplementary video legend**

**Supplementary video 1:** Real-time amplification curves using optical segmentation and corresponding images for both MRSA positive and MRSA negative samples with 100k copies of internal amplification control (IAC), and a true negative.
